# Cancer in the offspring of survivors of childhood leukaemia and non-Hodgkin lymphomas.

**DOI:** 10.1038/bjc.1995.259

**Published:** 1995-06

**Authors:** M. M. Hawkins, G. J. Draper, D. L. Winter

**Affiliations:** Childhood Cancer Research Group, University of Oxford, UK.

## Abstract

Understanding the extent to which childhood leukaemia and non-Hodgkin lymphomas are heritable is important to the survivors of these diseases, their families and clinicians who provide genetic counselling. Such understanding is also relevant to the possibility raised by Gardner et al. (1990, Br. Med. J., 300, 423-429) that paternal preconception irradiation may be an aetiological factor in these diseases. No malignant neoplasm was diagnosed among 382 offspring of survivors of childhood leukaemia and non-Hodgkin lymphoma followed up for a median period of 5.8 years, the largest available cohort of such offspring. These data indicate that it is unlikely that the risk of a malignant neoplasm occurring in the offspring exceeds eight times that expected in the general population. Similarly, the risk of leukaemia and non-Hodgkin lymphoma among offspring is unlikely to exceed 21 times that expected. The proportion of survivors of childhood leukaemia and non-Hodgkin lymphoma with the heritable form of these diseases is unlikely to exceed 5%, assuming an autosomal dominant pattern of transmission, with penetrance of at least 70% and that all heritable cases develop by age 15 years. The best (i.e. at present most likely) estimates of these risks are of course much lower. There was no evidence of an excess of congenital abnormalities among the offspring and the sex ratio was similar to that expected from the general population.


© 1995* 0007-0920/95 $12.00

### Cancer in the offspring of survivors of childhood leukaemia and non-Hodgkin lymphomas†

MM Hawkins, GJ Draper and DL Winter

*Childhood Cancer Research Group, University of Oxford, 57 Woodstock Road, Oxford OX2 6HJ, UK.*

**Summary** Understanding the extent to which childhood leukaemia and non-Hodgkin lymphomas are heritable is important to the survivors of these diseases, their families and clinicians who provide genetic counselling. Such understanding is also relevant to the possibility raised by Gardner *et al.* (1990, *Br. Med. J.*, **300**, 423-429) that paternal preconception irradiation may be an aetiological factor in these diseases. No malignant neoplasm was diagnosed among 382 offspring of survivors of childhood leukaemia and non-Hodgkin lymphoma followed up for a median period of 5.8 years, the largest available cohort of such offspring. These data indicate that it is unlikely that the risk of a malignant neoplasm occurring in the offspring exceeds eight times that expected in the general population. Similarly, the risk of leukaemia and non-Hodgkin lymphoma among offspring is unlikely to exceed 21 times that expected. The proportion of survivors of childhood leukaemia and non-Hodgkin lymphoma with the heritable form of these diseases is unlikely to exceed 5%, assuming an autosomal dominant pattern of transmission, with penetrance of at least 70% and that all heritable cases develop by age 15 years. The *best* (i.e. at present most likely) estimates of these risks are of course much lower. There was no evidence of an excess of congenital abnormalities among the offspring and the sex ratio was similar to that expected from the general population.

**Keywords:** leukaemia; heredity; offspring; non-Hodgkin lymphomas; genetics

Knowledge of the frequency of occurrence of malignant neoplasms generally, and leukaemia and non-Hodgkin lymphomas in particular, among the offspring of survivors of leukaemia and non-Hodgkin lymphomas diagnosed in childhood is important in that it will provide clarification of the extent to which these diseases are heritable. Such information is of great importance to survivors of these diseases, their families and the clinicians who provide genetic counselling. This information is also relevant to the question of whether paternal preconception irradiation is involved in the aetiology of childhood leukaemia and non-Hodgkin lymphomas (Gardner *et al.*, 1990).

Malignant neoplasms occur rarely in childhood – there are 1200 newly diagnosed patients aged under 15 each year in Britain. During recent decades there have been considerable improvements in the proportion of patients surviving at least 5 years from diagnosis, and about 90% of such survivors appear cured (Robertson *et al.*, 1994). Until recently there were insufficient patients surviving to provide an adequate number of offspring to estimate their risk of developing malignant neoplasms. As a consequence there have been few previous studies concerned with the occurrence of malignant neoplasms among the offspring of survivors of childhood cancer. We report on the risk of occurrence of malignant neoplasms within the largest available cohort of offspring of survivors of childhood leukaemia and non-Hodgkin lymphoma.

### Materials and methods

Using the population-based National Registry of Childhood Tumours, we identified 5227 patients diagnosed with cancer below age 15 years in Britain since 1940 who were born before 1969 and who were still alive. A postal questionnaire was sent mainly to the general practitioners of survivors. For the 959 survivors treated since 1976 we initially attempted to identify a hospital consultant who was responsible for the clinical follow-up of the patient. If we were unable to locate such a consultant, or if he or she did not return a satisfactorily completed questionnaire then we approached the general practitioner of the patient. The questionnaire was mainly concerned with outcomes of pregnancies and the health of offspring of survivors. In this initial paper we focus on the occurrence of malignant neoplasms among the offspring of the subgroup of survivors diagnosed with leukaemia or non-Hodgkin lymphoma in childhood. In subsequent papers we shall address the question of the heritability of other specific types of childhood cancer and also whether there is evidence of therapy-related germ cell mutagenesis from consideration of the pregnancies and offspring of survivors of all types of childhood cancer.

For the purposes of the present analysis we combined information relating to the offspring of survivors of childhood leukaemia and non-Hodgkin lymphomas because the numbers were too small to allow a meaningful analysis by more specific diagnostic categories. This combined diagnostic category comprises a constellation of diseases and, although some cases of non-Hodgkin lymphoma may truly be the same disease as some leukaemias, in general the heredity of these different diseases may be different.

Using registration rates of malignant neoplasms occurring throughout England and Wales, and provided on magnetic tape by the Office of Population Censuses and Surveys, we have calculated numbers of incident malignant neoplasms expected among the offspring using population rates stratified by sex, age and calender period (both in 5 year intervals) and multiplying the rate by the corresponding person-years at risk. Similar methods were used to obtain the expected number of deaths from all causes and also the expected number of deaths from each of congenital abnormalities and malignant neoplasms. Deaths from congenital abnormalities were considered because an excess of such deaths might give some clue concerning inherited susceptibility. From the observed and expected numbers of a specific outcome it is possible, using standard Poisson assumptions, to obtain an upper (one-sided) 95% confidence bound on the relative risk of the specific outcome among the offspring of survivors as compared with the risk in the general population (Pearson and Hartley, 1976).

Most human cancers can occur in individuals who are genetically predisposed. The most readily detected pattern of genetic susceptibility involves autosomal dominant

†This paper is dedicated to the memory of Emma Williamson
*Crown Copyright
Correspondence: MM Hawkins
Received 6 September 1994; revised 5 December 1994; accepted 27 January 1995

inheritance with high penetrance, particularly if the cancer appears at an unusually early age (Knudson, 1993). For the majority of such syndromes identified, the molecular mechanism involves tumour-suppressor genes (Knudson, 1993). There is evidence that this mechanism is involved in the development of leukaemias associated with the Li–Fraumeni syndrome (Malkin, 1993).

We investigate the implications of assuming that a proportion of leukaemia and non-Hodgkin lymphoma survivors have a heritable form of these diseases and that these are transmitted as an autosomal dominant with specified penetrance. In the statistical appendix we develop an expression for the probability of the observed data on the offspring under these assumptions. Using this expression we may obtain an upper (one-sided) confidence bound for the proportion of survivors with the heritable form of leukaemia or non-Hodgkin lymphoma.

We compared the observed numbers of serious (ie potentially lethal or handicapping congenital abnormalities occurring among offspring with those expected from some of the most reliable sources of birth prevalence available in Britain, and compiled by Leck (1994). The sex ratio observed among the offspring was compared with that expected from the sex ratio observed in the general population of England and Wales (Office of Population Censuses and Surveys, 1991).

### Results

There were 885 survivors of leukaemia or non-Hodgkin lymphoma eligible for the study in that they satisfied the criteria given above. For 852 of these we identified either a current general practitioner or a hospital consultant and sent out a questionnaire. A questionnaire was returned completed on 737, a response rate of 86.5%. For 51 of the survivors for whom no questionnaire was returned we had a questionnaire from a previous similar postal survey. From the totality of questionnaires available there was evidence of 401 live births, though in some instances the information provided was inadequate. After again contacting all of the general practitioners responsible for survivors for whom there was inadequate information on offspring we ultimately obtained sufficient information to include 382 offspring in a cohort analysis. For inclusion in the cohort we required of each offspring: date of birth, sex and an 'exit date', the last being a follow-up date for which it was known whether the offspring had ever been diagnosed with a malignant neoplasm. A total of 2776 person–years of follow-up were accrued on the cohort of offspring. The mean and median follow-up periods (i.e. ages at last follow-up) were 7.3 and 5.8 years respectively.

No malignant neoplasms occurred among the offspring. The expected number based on rates of these diseases in the general population was 0.36, which gives an upper (one-sided) 95% confidence bound on the relative risk of malignant neoplasms among the offspring of 8.4. The expected number of leukaemias and non-Hodgkin lymphomas was 0.14: this gives an upper (one-sided) 95% confidence bound on the relative risk of leukaemia or non-Hodgkin lymphomas among the offspring of 21.4. The latter confidence bound is high but is determined by the small number of offspring available and the rarity with which these diseases are expected to occur.

In Table I we give the upper (one-sided) 95% and 99% confidence bounds for the proportion of survivors with heritable leukaemia or non-Hodgkin lymphoma on the assumption that the mode of inheritance is that of a simple dominant gene with the indicated level of penetrance. Separate confidence bounds are given depending on whether it is assumed that all heritable cases will appear by age 15 years or by age 50 years. If we assume that all such cases manifest by age 15, then, if the diseases had a penetrance of at least 0.7, the proportion of heritable cases among the survivors is unlikely to exceed 5%.

In Table II we give the observed and expected numbers of deaths among the offspring. There was one death from a

**Table I** Upper (one-sided) 95% and 99% confidence bounds for the proportion of survivors with heritable leukaemia or non-Hodgkin lymphoma

| *Penetrance* | *Upper 95% confidence bound* | *Upper 99% confidence bound* |
|---|---|---|
| *a) Assuming all heritable cases appear by age 15* | | |
| 1.0 | 0.04 | 0.06 |
| 0.9 | 0.04 | 0.06 |
| 0.8 | 0.04 | 0.07 |
| 0.7 | 0.05 | 0.08 |
| 0.6 | 0.06 | 0.09 |
| 0.5 | 0.07 | 0.10 |
| 0.4 | 0.08 | 0.12 |
| 0.3 | 0.10 | 0.16 |
| 0.2 | 0.15 | 0.24 |
| *b) Assuming all heritable cases appear by age 50* | | |
| 1.0 | 0.12 | 0.19 |
| 0.9 | 0.13 | 0.20 |
| 0.8 | 0.15 | 0.23 |
| 0.7 | 0.17 | 0.26 |
| 0.6 | 0.19 | 0.30 |
| 0.5 | 0.23 | 0.36 |
| 0.4 | 0.29 | 0.45 |
| 0.3 | 0.38 | 0.59 |
| 0.2 | 0.57 | 0.89 |

**Table II** Observed and expected deaths among offspring of survivors of leukaemia and non-Hodgkin lymphomas

| *Cause of death* | *Observed No. (O)* | *Expected No. (E)* | *Relative risk (O/E)* | *95% confidence interval on relative risk* |
|---|---|---|---|---|
| Congenital abnormalities | 1 | 0.74 | 1.35 | (0.03, 7.54) |
| Malignant neoplasms | 0 | 0.15 | 0.00 | |
| Other causes | 6 | 2.61 | 2.30 | (0.84, 5.02) |
| Total | 7 | 3.51 | 2.00 | (0.80, 4.11) |

congenital abnormality: a tracheo-oesophageal fistula. None of the remaining six deaths was due to a neoplasm or congenital abnormality. Two deaths were due to pneumonia in offspring with pre-existing brain damage, three to complications of a premature delivery and one to a severe head injury; the circumstances in which this arose are not specified.

Investigation of the congenital abnormalities occurring among the offspring indicated that the observed frequency of serious abnormalities was broadly similar to that expected (Leck, 1994). However, there were four offspring, of different parents, who were diagnosed with pyloric stenosis. If we take the prevalence at birth as between 3 and 5 per 1000 total births (Leck, 1994) then the expected number lies between $382 \times 0.003 = 1.15$ and $382 \times 0.005 = 1.91$. Assuming underlying Poisson variability then the one-tailed $P$-values associated with these expected numbers are 0.03 and 0.13 respectively. Therefore, the observed number is not sufficiently discrepant from that expected to conclude that there is evidence of an excess.

Table III summarises the sex of offspring in relation to the sex of their parents. There was some evidence of an excess of male offspring born to female survivors when compared with the sex ratio in the general population ($P = 0.06$, two-tailed test) (Office of Population Censuses and Surveys, 1991). Although this excess was apparent among the offspring of both leukaemia and non-Hodgkin lymphoma survivors separately, it may well be due to chance, and further interpretation is not justified without independent confirmation. It is of interest that in a previous study Fraumeni (1964) reported that only 24 of 56 offspring of mothers diagnosed with leukaemia, mainly acute myeloid, were male.

### Discussion

Harnden (1985) has concluded that the evidence that inherited factors are important in the aetiology of leukaemia and lymphoma is not strong. In a recent review of the natural history of childhood acute leukaemia Greaves (1993) has concluded that: 'inherited abnormal alleles are likely to play a part in childhood leukaemia; their actual contribution may well be relatively small but is almost certainly underestimated at present'. The most direct way to estimate the extent of the inherited component of childhood leukaemia and non-Hodgkin lymphoma is to study the frequency of occurrence of these diseases among the offspring of patients who survive. This is not entirely without problems of interpretation since it might be that the group of patients who survive and produce offspring may contain a different proportion of heritable cases to those originally diagnosed. If we assume that such selection does not take place, then from Table I it is apparent that, assuming that all heritable cases occur by age 15 years, the proportion of survivors with the heritable form of leukaemia and non-Hodgkin lymphoma is unlikely to exceed 5%. This also assumes that the pattern of inheritance is that of an autosomal dominant gene with penetrance of at least 0.7.

A critical assumption underlying the derivation of the upper confidence bound on the proportion of heritable cases is that the age distribution for the heritable cases is proportionate to that for sporadic cases (see the statistical appendix). This is unlikely to be true. However, experience from retinoblastoma indicates that heritable cases develop at younger ages than sporadic cases (Draper *et al.*, 1992), and it is reasonable to assume the same would apply to heritable leukaemia and non-Hodgkin lymphomas. On this basis the upper confidence bound we have estimated will almost certainly be too large, although by how much is unknown because of uncertainty relating to the true age distribution of the heritable cases.

The heritability of childhood leukaemia and non-Hodgkin lymphoma has assumed considerable importance as an occupational and public health issue as a result of the suggestion that paternal preconceptual occupational exposure to radiation at the Sellafield nuclear reprocessing plant may be involved in the development of these diseases (Gardner *et al.*, 1990). Recently Doll *et al.* (1994) have cited our previous study of the risk of cancer among the 1348 offspring of survivors of childhood cancer (Hawkins *et al.*, 1989) in support of the conclusion that occupational exposure to radiation is not an aetiological factor. Our previous study included 86 survivors of leukaemia and non-Hodgkin lymphoma in childhood who produced 158 offspring, none of whom developed any malignant neoplasm (Hawkins *et al.*, 1989). All of the offspring included in that study are included in the present one, but with an extended period of follow-up. Broadly, the present study confirms and extends the evidence that inherited abnormal alleles do not appear to be important in the aetiology of childhood leukaemia and non-Hodgkin lymphomas under the assumed Mendelian model. However, less restrictive or more complicated modes of inheritance might be hypothesised which would require many

**Table III** Sex of offspring in relation to sex of survivor

| | *Sex of survivor* *Male* | *Female* | *Total* |
|---|---|---|---|
| Sex of offspring | | | |
| Male | 78 | 130 | 208 |
| Female | 79 | 95 | 174 |
| Total | 157 | 225 | 382 |

**Table IV** Previous studies of cancer in offspring of survivors of childhood or adolescent leukaemia or non-Hodgkin lymphoma (NHL)

| | *Numbers of offspring* *Leukaemia* | *NHL* | *Total* | *Total person–years* | *Average follow-up (nearest year)*[a] | *Cancers in offspring* |
|---|---|---|---|---|---|---|
| Mulvihill *et al.* (1987) | 28 | 140 | 168 | 1691 | 10 | Survivor–ALL Offspring–AL |
| Li *et al.* (1979) | ? | 59 | 59 | ? | ? | None |
| Nygaard *et al.* (1991) | 48 | 0 | 48 | 284 | 6 | None |
| Marradi *et al.* (1982) | 23 | 0 | 23 | ? | 5 | None |
| Green *et al.* (1989) | 22 | 0 | 22 | ? | 6 | None |
| Rokicka-Milewska, *et al.* (1986) | 7 | 0 | 7 | 25 | 4 | None |
| Total | 128 | 199 | 327 | | | |

ALL, acute lymphoblastic leukaemia; AL, acute leukaemia not otherwise specified; NHL, non-Hodgkin lymphoma; [a]Average, mean or median.

more data to test adequately. The present study strengthens the case for the interpretation which Doll and colleagues made relating to our earlier study.

The previous studies of the occurrence of cancer in the offspring of survivors of childhood or adolescent leukaemia or non-Hodgkin lymphomas are summarised in Table IV. In total there were 327 offspring reported, among whom one malignant neoplasm has been observed. A female survivor of acute lymphoblastic leukaemia who was treated with multiple chemotherapy produced a daughter who apparently died of acute leukaemia (not otherwise specified) at 6 months of age.

In conclusion, there are at present 709 known offspring of survivors of childhood and adolescent leukaemia or non-Hodgkin lymphoma, 382 from our study and 327 from previous studies, who have been followed up on average to at least 6 years of age. One of these offspring appears to have developed a disease similar to that successfully treated in her mother. Unfortunately, the original investigators were unable to contact the survivor concerned and are unable to confirm the clinical details of the disease in the offspring (Mulvihill *et al.*, 1987). From our study there is no evidence of an increased risk of malignant neoplasms among offspring as compared with the general population; it is unlikely that the risk is greater than the 8-fold expected. Furthermore, the proportion of survivors of childhood leukaemia and non-Hodgkin lymphomas with the heritable form of these diseases is unlikely to exceed 5%, assuming an autosomal dominant mode of transmission with penetrance of 70–100%. More precise estimates of the heritable component of these diseases could be obtained by pooling the original data from this and previous studies and using similar methods of analysis to those adopted above.

The practical message to be derived from this and previous studies is that, although any detailed estimates of heritability depend on a number of unverified assumptions, and although a degree of caution is necessary until more offspring have been followed up, the empirically observed risks to offspring are small.

**Acknowledgements**

Particular thanks are due to Lis Mowat, Hazel Burton and Michael Potok for their considerable contribution towards this study. We are very grateful to Professors AW Craft, JM Chessells, OB Eden, DG Harnden, JS Malpas and HB Marsden and Drs CC Bailey, JR Mann PH Morris Jones and MCG Stevens for their continuing advice and support. We also thank the many consultants who gave access to their medical records. The research is mainly funded by the Cancer Research Campaign with a supplementary grant from the Leukaemia Research Fund. The Childhood Cancer Research Group is supported by the Department of Health and the Scottish Home and Health Department. We are also grateful to the United Kingdom Children's Cancer Study Group; the Office of Population Censuses and Surveys; the Information Services Division of the Common Services Agency of the Scottish Health Service; the regional cancer registries; the Registrar General for Scotland; and the NHS Central Registers, Southport and Edinburgh.

**References**

DOLL R, EVANS HJ AND DARBY SC. (1994). Paternal exposure not to blame. *Nature*, **367**, 678–680.

DRAPER GJ, SANDERS BM, BROWNBILL PA AND HAWKINS MM. (1992). Patterns of risk of hereditary retinoblastoma and applications to genetic counselling. *Br. J. Cancer*, **66**, 211–19.

FRAUMENI JF. (1964). Sex ratio of children born of leukaemic mothers. *Pediatrics*, **31**, 587–89.

GARDNER MJ, SNEE MP, HALL AJ, POWELL CA, DOWNES S AND TERRELL JD. (1990). Results of case–control study of leukaemia and non-Hodgkin lymphoma among young people near Sellafield nuclear plant in West Cumbria. *Br. Med. J.*, **300**, 423–429.

GREAVES M. (1993). A natural history for pediatric acute leukemia. *Blood*, **82**, 1043–1051.

GREEN DM, HALL B AND ZEVON MA. (1989). Pregnancy outcome after treatment for acute lymphoblastic leukemia during childhood or adolescence. *Cancer*, **64**, 2335–2339.

HARNDEN DG. (1985). Inherited factors in leukaemia and lymphoma. *Leukaemia Res.*, **9**, 705–07.

HAWKINS MM, DRAPER GJ AND SMITH RA. (1989). Cancer among 1,348 offspring of survivors of childhood cancer. *Int. J. Cancer*, **43**, 975–978.

KNUDSON AG. (1993). Antioncogenes and human cancer. *Proc. Natl Acad. Sci. USA*, **90**, 10914–921.

LECK I. (1994). Structural birth defects. In *The Epidemiology of Childhood Disorders*, Pless IB. (ed.) pp. 66–117. Oxford University Press: Oxford.

LI FP, FINE W, JAFFE N, HOLMES FF AND HOLMES GE. (1979). Offspring of patients treated for cancer in childhood. *J. Natl Cancer Inst.*, **62**, 1193–1197.

MALKIN D. (1993). p53 and the Li–Fraumeni syndrome. *Cancer Genet. Cytogenet*, **66**, 83–92.

MARRADI P, SCHAISON G, ALBY N, BERGER R, JACQUILLAT C AND BOIRON M. (1982). Les enfants nés de parents leucémiques. A propos de 23 enfants. (Children from leukaemic parents; abstract in English). *Nouv. Rev. Fr. Hematol.*, **24**, 75–80.

MULVIHILL JJ, MYERS MH, CONNELLY RR, BYRNE J, AUSTIN DF, BRAGG K, COOK JW, HASSINGER DD, HOLMES FF, HOLMES GF, KRAUSS MR, LATOURETTE HB, MEIGS JW, NAUGHTON MD, STEINHORN SC, STRONG LC, TETA MJ AND WEYER PJ. (1987). Cancer in offspring of long-term survivors of childhood and adolescent cancer. *Lancet*, **i**, 813–817.

NYGAARD R, CLAUSEN N, SIIMES MA, MARKY I, SKJELDESTAD FE, KRISTINSSON JR, VUORISTO A, WEGELIUS R AND MOE PJ. (1991). Reproduction following treatment for childhood leukemia: a population-based prospective cohort study of fertility and offspring. *Med. Pediatr. Oncol.*, **19**, 459–466.

OFFICE OF POPULATION CENSUSES AND SURVEYS. (1993). *Birth Statistics England and Wales 1991*, Series FM1, No. 9. HMSO: London.

PEARSON ES AND HARTLEY HO. (1976). *Biometrika Tables for Statisticians*, Vol. 1, table 40. Biometrika Trust: London.

ROBERTSON CM, HAWKINS MM AND KINGSTON JE. (1994). Late deaths and survival after childhood cancer: implications for cure. *Br. Med. J.*, **309**, 162–166.

ROKICKA-MILEWSKA R, DERULSKA D, ARMATA J, BALWIERZ W, BOGUSLAWSKA-JAWORSKA J, CYKLIS R, DUCZMAL B, MICHAELEWSKA D, NEWECKA T, OCHOCKA M, RADWANSKA U, RODZIEWICZ B AND SONTA-JAKIMEZYK D. (1986). Children cured of acute lymphoid leukemia. Long-term follow-up studies, including progeny. *Am. J. Pediatr. Hematol. Oncol.*, **8**, 208–212.

**Statistical appendix**

As explained in the text we have used the results of this study to examine the possibility that some cases of leukaemia and non-Hodgkin lymphoma are heritable. Specifically, we have considered the hypothesis that in a fraction $\lambda$ of cases these diseases can be transmitted in a simple autosomal dominant pattern with specified penetrance. With these assumptions a one-sided upper confidence limit for $\lambda$ was calculated using varying assumptions about the penetrance and the age distribution of heritable cases.

For the general population we denote by $q(t)$ the probability of developing leukaemia or non-Hodgkin lymphoma by age $t$. We assume also that all heritable cases that manifest the disease do so by age $T$ and that for these cases the age distribution is proportionate to that for similar malignant neoplasms in the general population: then for $t \leqslant T$ the conditional probability of the disease occurring by age $t$ is $p(t)$, where $p(t) = q(t)/q(T)$; for $t > T, p(t) = 1$. The absolute probability of not manifesting the disease by age $t$, assuming a dominant pattern of inheritance is $[1-0.5 \times (\text{penetrance}) \times p(t)]$.

For a given family, denote the ages to which the offspring are followed up by $t_i (i = 1,2,\cdots)$. Then the probability of the observed offspring data is:

$$\prod_{\text{families}} \{\lambda\ [\text{prob (no offspring affected} \mid \text{survivor carries gene)}] + (1\text{-}\lambda)\ [\text{prob (no offspring affected} \mid \text{survivor does not carry gene)}]\}$$

$$= \prod_{\text{families}} \{\lambda \prod_{\substack{\text{offspring } (i)\\ \text{within family}}} [1\text{-}(0.5) \times (\text{penetrance}) \times p(t_i)] + (1-\lambda) \prod_{\substack{\text{offspring } (i)\\ \text{within family}}} [1-q(t_i)]\}$$

The $q(t)$ values were calculated from population rates of malignant neoplasms. For values of $t = 1,2,\cdots$, 15 years they were calculated using unpublished data from the National Registry of Childhood Tumours. For $t = 16,17,\cdots$, 50 years they were calculated from the Leukaemia Research Fund survey of leukaemia and lymphomas*.

The expression above has two parameters, $\lambda$ and the penetrance. In Table I we give the 95% and 99% upper (one-sided) confidence bounds on $\lambda$ for varying values of the penetrance. These confidence bounds are evaluated by finding the values $\lambda$, $\lambda_{95}$ and $\lambda_{99}$, say, such that, for a specified value of the penetrance, the probability of the observed data is 0.05 or 0.01 respectively. In Table I (a) it is assumed that all heritable cases occur by age 15; in Table I (b) they are assumed to occur by age 50.

*LEUKAEMIA RESEARCH FUND. (1990). *Leukaemia and Lymphoma: an Atlas of Distribution within Areas of England and Wales, 1984–1988*. Leukaemia Research Fund: London.


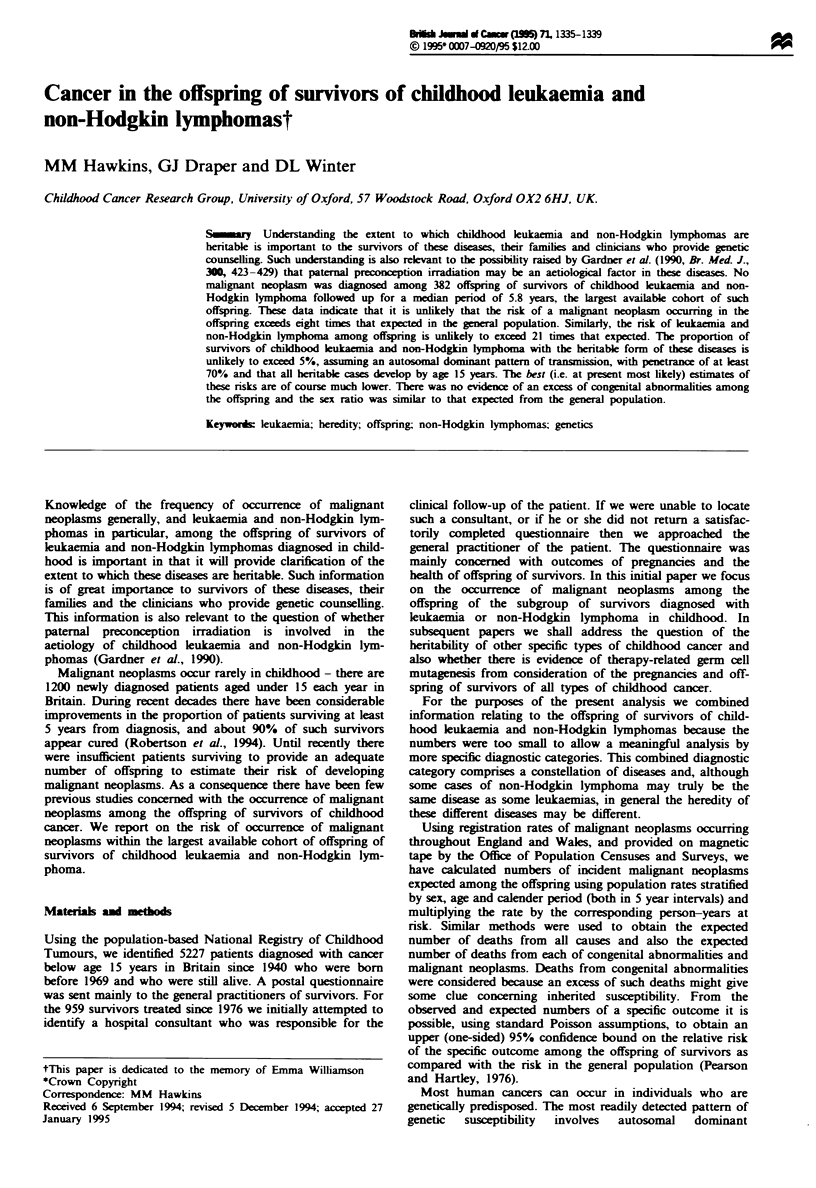

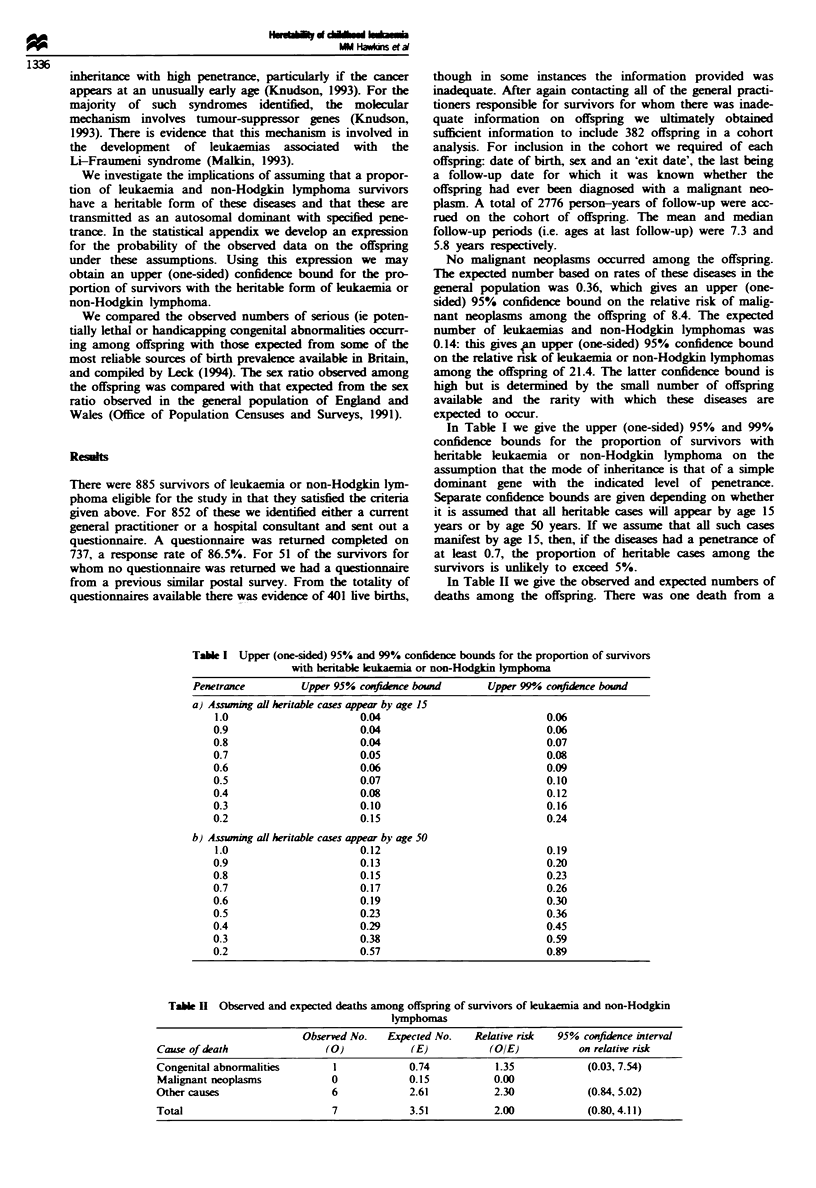

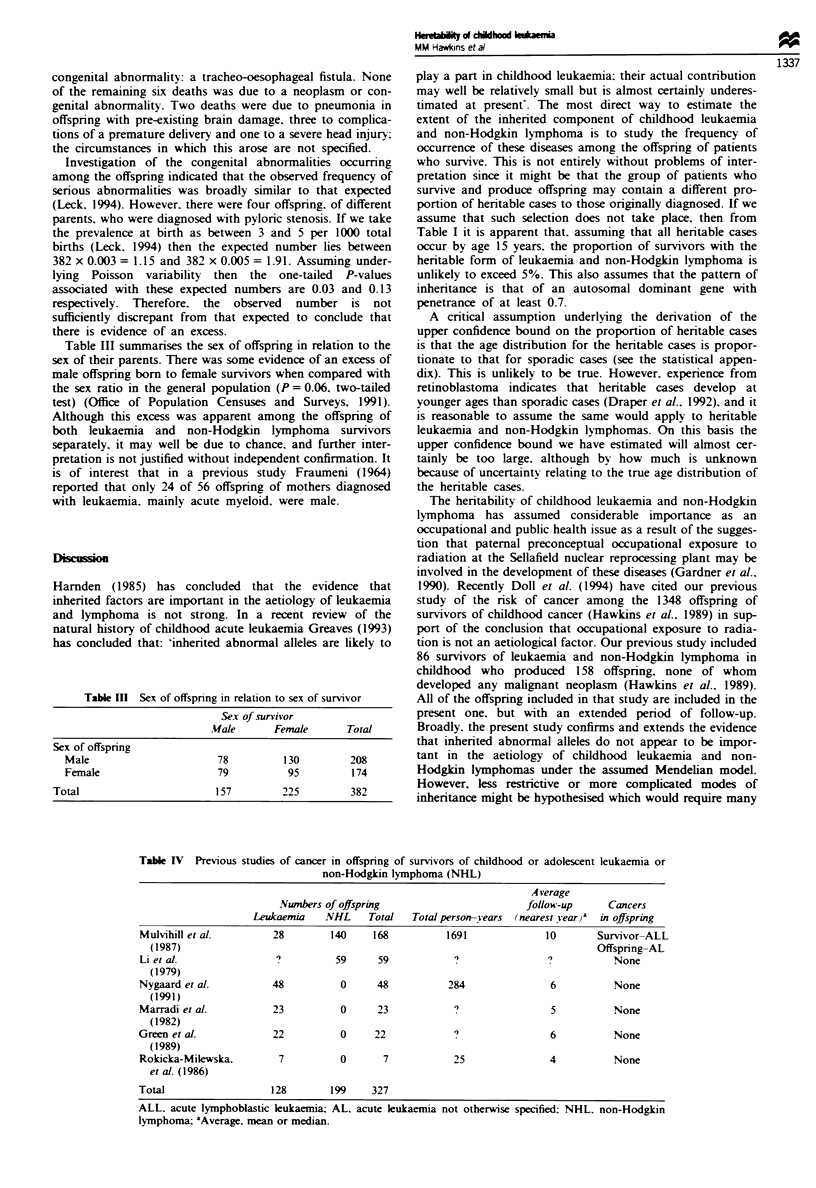

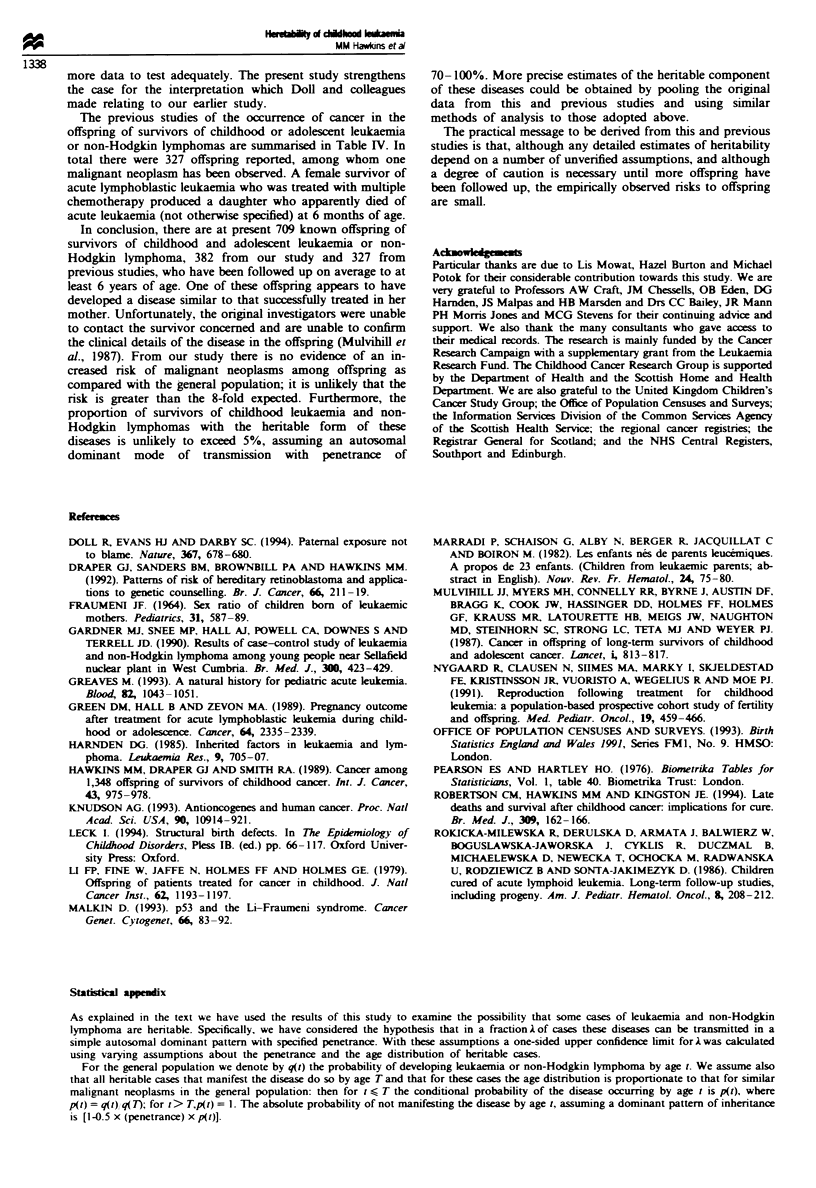

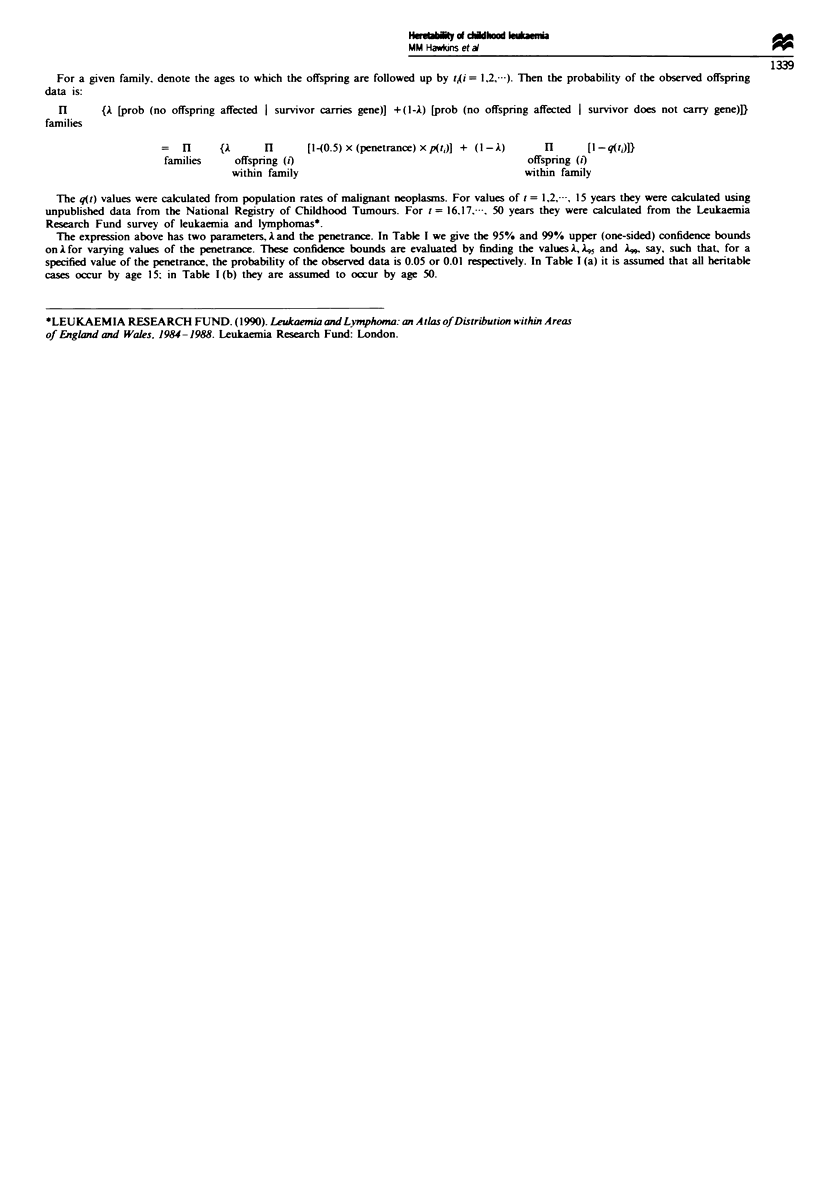


## References

[OCR_00503] Doll R., Evans H. J., Darby S. C. (1994). Paternal exposure not to blame.. Nature.

[OCR_00507] Draper G. J., Sanders B. M., Brownbill P. A., Hawkins M. M. (1992). Patterns of risk of hereditary retinoblastoma and applications to genetic counselling.. Br J Cancer.

[OCR_00510] FRAUMENI J. F. (1964). SEX RATIO OF CHILDREN BORN OF LEUKEMIC MOTHERS.. Pediatrics.

[OCR_00517] Gardner M. J., Snee M. P., Hall A. J., Powell C. A., Downes S., Terrell J. D. (1990). Results of case-control study of leukaemia and lymphoma among young people near Sellafield nuclear plant in West Cumbria.. BMJ.

[OCR_00522] Greaves M. (1993). A natural history for pediatric acute leukemia.. Blood.

[OCR_00524] Green D. M., Hall B., Zevon M. A. (1989). Pregnancy outcome after treatment for acute lymphoblastic leukemia during childhood or adolescence.. Cancer.

[OCR_00531] Harnden D. G. (1985). Inherited factors in leukaemia and lymphoma.. Leuk Res.

[OCR_00533] Hawkins M. M., Draper G. J., Smith R. A. (1989). Cancer among 1,348 offspring of survivors of childhood cancer.. Int J Cancer.

[OCR_00538] Knudson A. G. (1993). Antioncogenes and human cancer.. Proc Natl Acad Sci U S A.

[OCR_00549] Li F. P., Fine W., Jaffe N., Holmes G. E., Holmes F. F. (1979). Offspring of patients treated for cancer in childhood.. J Natl Cancer Inst.

[OCR_00554] Malkin D. (1993). p53 and the Li-Fraumeni syndrome.. Cancer Genet Cytogenet.

[OCR_00556] Marradi P., Schaison G., Alby N., Berger R., Jacquillat C., Boiron M. (1982). Les enfants nés de parents leucémiques. A propos de 23 enfants.. Nouv Rev Fr Hematol.

[OCR_00565] Mulvihill J. J., Myers M. H., Connelly R. R., Byrne J., Austin D. F., Bragg K., Cook J. W., Hassinger D. D., Holmes F. F., Holmes G. F. (1987). Cancer in offspring of long-term survivors of childhood and adolescent cancer.. Lancet.

[OCR_00570] Nygaard R., Clausen N., Siimes M. A., Márky I., Skjeldestad F. E., Kristinsson J. R., Vuoristo A., Wegelius R., Moe P. J. (1991). Reproduction following treatment for childhood leukemia: a population-based prospective cohort study of fertility and offspring.. Med Pediatr Oncol.

[OCR_00588] Robertson C. M., Hawkins M. M., Kingston J. E. (1994). Late deaths and survival after childhood cancer: implications for cure.. BMJ.

[OCR_00595] Rokicka-Milewska R., Derulska D., Armata J., Balwierz W., Boguslawska-Jaworska J., Cyklis R., Duczmal B., Michalewska D., Newecka T., Ochocka M. (1986). Children cured of acute lymphoid leukemia. Long-term follow-up studies, including progeny.. Am J Pediatr Hematol Oncol.

